# Plastome Rearrangements in the “*Adenocalymma-Neojobertia*” Clade (Bignonieae, Bignoniaceae) and Its Phylogenetic Implications

**DOI:** 10.3389/fpls.2017.01875

**Published:** 2017-11-01

**Authors:** Luiz H. M. Fonseca, Lúcia G. Lohmann

**Affiliations:** Laboratorio de Sistemática Vegetal, Departamento de Botânica, Instituto de Biocências, Universidade de São Paulo, São Paulo, Brazil

**Keywords:** cp genome, genomic rearrangments, DNA sequence alignment, phylogenomics, plastid primers

## Abstract

The chloroplast is one of the most important organelles of plants. This organelle has a circular DNA with approximately 130 genes. The use of plastid genomic data in phylogenetic and evolutionary studies became possible with high-throughput sequencing methods, which allowed us to rapidly obtain complete genomes at a reasonable cost. Here, we use high-throughput sequencing to study the “*Adenocalymma*-*Neojobertia*” clade (Bignonieae, Bignoniaceae). More specifically, we use Hi-Seq Illumina technology to sequence 10 complete plastid genomes. Plastomes were assembled using selected plastid reads and *de novo* approach with SPAdes. The 10 assembled genomes were analyzed in a phylogenetic context using five different partition schemes: (1) 91 protein-coding genes (“coding”); (2) 76 introns and spacers with alignment manually edited (“non-coding edited”); (3) 76 non-coding regions with poorly aligned regions removed using T-Coffee (“non-coding filtered”); (4) 91 coding regions plus 76 non-coding regions edited (“coding + non-coding edited”); and, (5) 91 protein-coding regions plus the 76 filtered non-coding regions (“coding + non-coding filtered”). Fragmented regions were aligned using Mafft. Phylogenetic analyses were conducted using Maximum Likelihood (ML) and Bayesian Criteria (BC). The analyses of the individual plastomes consistently recovered an expansion of the Inverted Repeated (IRs) regions and a compression of the Small Single Copy (SSC) region. Major genomic translocations were observed at the Large Single Copy (LSC) and IRs. ML phylogenetic analyses of the individual datasets led to the same topology, with the exception of the analysis of the “non-coding filtered” dataset. Overall, relationships were strongly supported, with the highest support values obtained through the analysis of the “coding + non-coding edited” dataset. Four regions at the LSC, SSC, and IR were selected for primer development. The “*Adenocalymma*-*Neojobertia*” clade shows an unusual pattern of plastid structure variation, including four major genomic translocations. These rearrangements challenge the current view of conserved plastid genome architecture in terms of gene order. It also complicates both genomic assemblies using reference genomes and sequence alignments using whole plastomes. Therefore, strategies that employ *de novo* assemblies and manual evaluation of sequence alignments are required to prevent assembly and alignment errors.

## Introduction

The plastome is the portion of the plant genome that contains all the genetic information included in the chloroplast (Bock, 2007). The chloroplast is an organelle of prokaryotic origin with a crucial role in photosynthesis and cell storage (Wise, 2006). It contains the biochemical machinery necessary to replicate its own genome, transcribe genes, and translate those genes into proteins (Wise, 2006). Plastomes have a circular genome of double-stranded DNA that ranges from 72 to 217 kb in flowering plants (Chumley et al., 2006), with approximately 130 genes (Sugiura, 1992, 1995). Genes found in the plastomes encode the core proteins of photosynthetic complexes, including Photosystem I and II, Cytochrome b_6_f, NADH dehidrogenase, ATP synthase and RUBISCO (Grenn, 2011). Chloroplast genomes typically include a quadripartite structure that consists of a small single copy region (SSC) with approximately 16–27 kb, a large single copy region (LSC) with approximately 80–90 kb, and a pair of inverted repeats (IRs) with approximately 20 to 28 kb each. Expansions and contractions of the IRs, as well as gene and intron losses have been documented in Angiosperms (Jansen et al., 2011; Liu et al., 2016). However, the overall chloroplast structure, gene content, and organization are thought to be highly conserved among flowering plants (Odintsova and Yurina, 2003; Wicke et al., 2011; Smith and Keeling, 2015; Reginato et al., 2016).

The conserved structure of the chloroplast genome facilitates PCR primer design and sequencing within Angiosperms (Small et al., 1998; Shaw et al., 2005, 2007). Efforts to resolve Angiosperm phylogenetic relationships at different taxonomic levels have traditionally used plastome coding and non-coding regions as sources of evidence (e.g., Soltis et al., 1999; Shaw et al., 2007). While these regions are very informative at higher taxonomic levels, they often lack sufficient variation to resolve relationships at the species or population levels, even when rapidly evolving non-coding DNA regions are considered (Small et al., 1998; Shaw et al., 2005, 2007). More recently, high-throughput sequencing methods have allowed researchers to rapidly obtain complete genomes at a reasonable cost (Cronn et al., 2008; Parks et al., 2009). These genomes have been used as basis for phylogenomic studies, leading to highly resolved and strongly supported phylogenies of several plant groups (Moore et al., 2010; Harrison et al., 2015; Wysocki et al., 2015).

The “*Adenocalymma-Neojobertia*” clade (Bignonieae, Bignoniaceae) is a lineage of lianas, shrubs and treelets that includes approximately 75 species. The genus exhibits substantial diversity in ecology, with species distributed from deciduous forests (e.g., Brazilian cerrados and caatingas) to tropical rain forests (e.g., Amazonia and Atlantic forest) (Lohmann and Taylor, 2014). A phylogenetic study of the whole tribe Bignonieae based on sequences of the nuclear intron *pep*C and the plastid gene *ndh*F was the only study to sample species of *Adenocalymma* Mart. ex Meisn. and *Neojobertia* Baill. to date (Lohmann, 2006). This study sampled 12 of the 75 species currently recognized and recovered a monophyletic “*Adenocalymma-Neojobertia*” clade (Lohmann, 2006). While generic-level clades were strongly supported in this study, resolution was week within the “*Adenocalymma-Neojobertia*” clade (Lohmann, 2006). Full plastomes generally include a high number of phylogenetic informative characters and can improve estimates of phylogenetic relationship at various taxonomic levels (e.g., Ma et al., 2014; Reginato et al., 2016). However, only the plastid genomes of *Tanaecium tetragonolobum* (Jacq.) L.G. Lohmann (NC_027955.1; Nazareno et al., 2015) and *Crescentia cujete* L. (KT182634.2; Moreira et al., 2016) are currently available for members of the plant family Bignoniaceae.

In this study, we used high-throughput sequencing technology to sequence ten complete plastomes of members of the “*Adenocalymma-Neojobertia*” clade in order to: (i) characterize the gene content, levels of sequence variation, and structure of plastomes within this clade; (ii) compare the plastomes of members of the “*Adenocalymma-Neojobertia*” clade with those available for other Bignoniaceae; (iii) explore the potential of genomic data for phylogenomic studies within the “*Adenocalymma-Neojobertia*” clade and the Bignoniaceae as a whole; and, (iv) identify informative markers for future species level phylogenetic studies.

## Materials and methods

### Taxon sampling and genome sequencing

We sampled 10 accessions of members of the “*Adenocalymma-Neojobertia*” clade, representing nine species of *Adenocalymma* plus one species of *Neojobertia* (NCBI accession numbers at Table 1). These species were selected in order to represent the breath of morphological diversity and geographical distribution within the clade. Total genomic DNA was extracted from silica-dried leaflets or herbarium specimens using the Invisorb® Spin Plant Mini Kit (Invitek, Berlin, Germany). Approximately 60 ng of leaf tissue were pulverized with Tissuelyzer® (Qiagen, Duesseldorf, Germany) for 3 min at 60 hz. Five micrograms of total DNA were fragmented using a Covaris S-series sonicator, generating DNA fragments of approximately 300 bp. Libraries were constructed using the NEBNext DNA Library Prep Master Mix Set and the NEBNext Multiplex oligos for Illumina (New England BioLabs Inc., Ipswich, MA) following the manufacturer's protocol. DNA library concentration was determined using the Kapa Library Quantification Kit (Kapa Biosystems Inc., Wilmington, MA) on an Applied Biosystems 7500 Real-Time PCR System. The final libraries were diluted to a concentration of 10 nM and put together in pools of 20 samples. Each pool of species was sequenced in a lane using pair-end (2 × 100) on an Illumina HiSeq 2000 system (Illumina Inc., San Diego, CA).

**Table 1:** Taxa, vouchers, collection sites, and accession numbers of *Adenocalymma* and *Neojobertia* specimens sampled.

**Taxa**	**Vouchers**	**Collection Sites**	**Accession**
*A. allamandiflorum*	B.M. Gomes 671 (SPF)	Brazil; Pará; Santarém	MG008307
*A. aurantiacum*	L.G. Lohmann 658 (SPF)	Brazil; Espírito Santo; Linhares	MG008308
*A. biternatum*	M.R. Pace 521 (SPF)	Peru; Loreto; Iquitos	MG008309
*A. bracteatum*	L.H.M. Fonseca 100 (SPF)	Brazil; Paraná; Jundiaí do Sul	MG008310
*A. cristicalyx*	L.G. Lohmann 705 (SPF)	Brazil; Paraíba; Alagoa Grande	MG008311
*A. nervosum*	L.H.M. Fonseca 262 (SPF)	Brazil; Minas Gerais; Catugi	MG008312
*A. pedunculatum*	L.H.M. Fonseca 267 (SPF)	Brazil; Minas Gerais; Diamantina	MG008313
*A. peregrinum*	L.H.M. Fonseca 444 (SPF)	Brazil; Goiás; São Jorge da Chapada	MG008314
*A. subspicatum*	R.G. Udulutsch 2758 (SPF)	Brazil; Ceará; Tianguá	MG008315
*N. candolleana*	L.G. Lohmann 363 (SPF)	Brazil; Bahia; Mucugê	MG008316

### Plastome assembly and annotation

Plastomes were assembled using the Fast-Plast pipeline (https://github.com/mrmckain/Fast-Plast; McKain and Wilson, unpublished). For each species, adaptors were removed and low quality sequences trimmed using Trimmomatic 0.35 (Bolger et al., 2014) with the SLIDINGWINDOW:10:20 and MINLEN:40 parameters. Trimmed reads were mapped against a database that included *C. cujete* L., *Erythranthe lutea* (L.) G.L. Nesom (NC_030212.1; Vallejo-Marín et al., 2016), *Olea europaea* L. (NC_013707.2; Messina, unpublished), *Sesamum indicum* L. (NC_016433.2; Yi and Kim, 2012), *Salvia miltiorhiza* Bunge (NC_020431.1; Qian et al., 2013), and *T. tetragonolobum* (Jacq.) L.G. Lohmann using Bowtie2 2.1.0 (Langmead and Salzberg, 2012) with the default parameters. Mapped reads were assembled into contigs using SPAdes 3.1.0 (Bankevich et al., 2012) with k-mer sizes of 55 and 87, using the “only-assembler” option. Resulting contigs were assembled with the software afin (https://bitbucket.org/afinit/afin nit/afin) and the default parameters -l 50, -f 0.1, -d 100, -x 100, and -i 2. For species for which it was harder to obtain comprehensive contigs, we tested different values for maximum percentage of mismatches (-g), and minimum overlap of contig (-p) parameters. For some species, the *de novo* assembly returned a large contig that contained the complete plastome. These contigs were checked and finalized with Geneious 9.0.2 (Kearse et al., 2012). The plastome assembly was verified through a coverage analysis conducted in Jellyfish 2.1.3 (Marçais and Kingsford, 2011). The estimate of 25-mer abundance was used to map a 25-bp sliding window of coverage across the plastome of each species.

Plastome annotation was initially conducted in DOGMA (Wyman et al., 2004). These annotations were checked in Geneious 9.0.2 using *O. europaea* and *Solanum lycopersicum* L. (NC_007898.3; Daniell et al., 2006) as references. Promising open reading frames at non-coding regions were verified with BLAST (Altschul et al., 1990) available at NCBI (https://www.ncbi.nlm.nih.gov/). Maps of the annotated plastomes were created using OGDRAW (Lohse et al., 2007). We characterized the overall plastome structure, gene content, and general gene information of the 10 species sampled and compared our results with the information available for two other Bignoniaceae (i.e., *C. cujete* and *T. tetragonolobum*), and one Oleaceae (i.e., *O. europaea*). Points of potential rearrangements and junctions between the IRs, the LSC, and SSC were tested iteratively using afin (https://bitbucket.org/afi nit/afin), and checked with PCR amplifications and electrophoresis. Coverage values for these regions were also assessed.

### Phylogenetic analyses

We used the LSC, SSC and one IR to infer the phylogenetic tree of the “*Adenocalymma-Neojobertia*” clade. We excluded one IR to avoid duplication of data. We used three chloroplast genomes of members of the Lamiales (*C. cujete, O. europaea*, and *T. tetragonolobum*) as outgroups. Pseudogenes and its orthologous were treated as non-coding regions. Genes with overlapping portions were treated as neighbors to avoid character duplication.

For the phylogenetic analyses, annotated plastomes were fragmented into coding and non-coding regions, excluding regions smaller than 50 bp. The retained regions were grouped by sequence similarity (with a threshold of 65% of global similarity and default alignment costs) using the annotated plastome of *Adenocalymma biternatum* (A. Samp.) L.G. Lohmann as reference and considering the pool of regions for all species. Plastome partitioning and sequence grouping was conducted using the R package Biostrings (R Development Core Team, 2017; Pagès et al., unpublished). Coding regions were aligned with MAFFT 7 (Katoh and Standley, 2013) using the G-INS-i 1,000 strategy, while non-coding regions were aligned using the E-INS-i 1,000 strategy. We removed poorly aligned regions of the coding and non-coding alignments using GBlocks (Castresana, 2000) default settings in order to circumvent homology assessment problems due to random similarity of sequences or indels. Alignments of non-coding regions with rearrangements were edited manually or misaligned sequences were removed using the outlier search option implemented in T-Coffee (Notredame et al., 2000). This was necessary since GBlocks is not able to recognize rare outlier sequences (Castresana, 2000). Three different partition schemes were built as follows: (1) 91 coding regions (“coding”); (2) 76 introns and spacers with alignment edited by hand (“non-coding edited”); and (3) 76 non-coding regions with poorly aligned sequences removed with T-Coffee (“non-coding filtered”). Combined datasets were also analyzed as follows: (4) 91 coding regions plus 76 non-coding regions (“coding + non-coding edited”); and (5) 91 coding regions plus the 76 filtered non-coding regions (“coding + non-coding filtered”). The five datasets were compared based on tree topology and node support.

All phylogenetic analyses were performed with Maximum Likelihood (ML) using RAxML 8.2.9 (Stamatakis, 2014), and Bayesian Criteria (BC) using MrBayes 3.2 (Ronquist et al., 2011). ML node support was estimated through a rapid bootstrap analysis with 1,000 replicates. BC were run using uniform priors and two independent runs of 10 million generations with four chains per run, sampling trees every 1,000 generations. BC support was estimated using posterior probabilities. For BC, chain convergence and stationarity were assessed using the R package Coda (R Development Core Team, 2017; Plummer et al., unpublished) by visually examining plots of parameter values and log-likelihood against the number of generations. For Bayesian analysis we employed the reversible jump strategy (Ronquist et al., 2011), which does not require the establishment of evolutionary models or partition schemes *a priori*. For ML the GTRCAT evolutionary model was used (Stamatakis, 2014), avoiding pre-defined partitions.

### Identification of markers for species level phylogenetic studies

Among the 76 introns and spacers recovered, we retained the 31 regions that were recombination free and with suitable length for PCR amplification (amplicons with size between 500 and 1,100 bp). These partitions were analyzed to identify highly informative regions that may serve as useful markers for future species level phylogenetic analyses. ML trees were inferred for each of the 31 partitions using RAxML 8.2.9 and the GTRCAT evolutionary model. For each partition, alignment length, variable sites, topological distance, and branch length distance (Kendall and Colijn, 2016) were estimated. Metrics were computed using the R packages (R Development Core Team, 2017) Ape (Paradis et al., 2004) and Treescape (Jombart et al., unpublished). Partitions were ranked using standardized values of the number of informative characters, as well as the topological and branch length distances between the tree derived from the analysis of each partition and the best tree estimated in this study (i.e., the tree derived from the analysis of the “coding + non-coding edited” dataset; see results). All metrics were computed for the “*Adenocalymma-Neojobertia*” clade exclusively. Four non-coding regions were selected with Geneious 9.0.2 for primer design.

## Results

### Plastome assembly

We sequenced the complete plastomes of 10 species of the “*Adenocalymma-Neojobertia*” clade using an Illumina HiSeq 2000 system, namely: *A. allamandiflorum* (Bureau ex K. Schum.) L.G. Lohmann, *A. aurantiacum* Udulutsch and Assis, *A. biternatum* (A. Samp.) L.G. Lohmann, *A. bracteatum* (Cham.) DC., *A. cristicalyx* (A.H Gentry) L.G. Lohmann, *A. nervosum* Bureau and K. Schum., *A. pedunculatum* (Vell.) L.G. Lohmann, *A. peregrinum* (Miers) L.G. Lohmann, *A. subspicatum* A.H. Gentry, and *N. candolleana* (Mart. ex DC.) Bureau and K. Schum. (Table 2). A minimum of 8,532,329, and a maximum of 30,862,472 paired end raw reads (with an average length of 101 bp) were generated for *N. candolleana* and *A. biternatum*, respectively. After mapping reads against the reference genomes of *C. cujete, T. tetragonolobum*, and *O. europaea*, a minimum of 239,286 reads and a maximum of 762,288 reads were retained for *A. subspicatum* and *A. bracteatum*, respectively. Plastome coverage ranged from 307.7 × to 964 × for *A. subspicatum* and *A. bracteatum*, respectively (Table 2). Junctions of the quadripartite structure and the regions with potential rearrangements were tested interactively and recovered in all combinations of parameters used. A high mean coverage value was obtained for all species, providing additional support for the plastome assemblies (Table 2). High coverage values were also observed at junctions of the quadripartite structure and regions with rearrangements. PCR and electrophoresis recovered the amplicons expected for each junction of the quadripartite structure and regions with potential rearrangements. No regions with low coverage (<20x) were recovered. The finished, high quality organelle genome sequences were used for downstream analyses.

**Table 2:** Summary of sequenced plastomes of *Adenocalymma* and *Neojobertia*.

**Species**	**No. of raw reads**	**No. of mapped reads**	**Plastome coverage (x)**	**Plastome length (bp)**	**LSC length (bp)**	**SSC length (bp)**	**IR length (bp)**	**GC content (%)**	**CDS**	**tRNA**	**rRNA**
*A. allamandiflorum*	14,546,510	284,977	364.4	157,952	84,668	12,804	30,240	38.2	85	37	8
*A. aurantiacum*	9,142,072	713,911	904.6	159,407	84,934	12,585	30,954	37.4	85	37	8
*A. biternatum*	30,862,472	501,539	645.2	157,025	84,059	12,723	30,097	38.2	84	37	8
*A. bracteatum*	17,633,356	762,288	964	159,725	85,665	12,632	30,835	38.3	85	37	8
*A. cristicalyx*	10,369,744	399,680	507.7	159,010	85,654	12,677	30,310	38	85	37	8
*A. nervosum*	19,780,568	432099	549.7	158,786	84,999	12,616	30,561	41.6	85	37	8
*A. pedunculatum*	13,881,445	305,321	390	158,103	85,043	12,730	30,147	37.6	85	37	8
*A. peregrinum*	18,872,525	506,711	643	159,187	85,106	12,804	30,614	38.3	84	37	8
*A. subspicatum*	11,761,741	239,286	307.7	157,089	84,219	12,765	30,084	37.7	85	37	8
*N. candolleana*	8,532,329	738,770	942	158,409	85,192	12,737	30,211	38.1	85	37	8

### Plastome features

**Figure 1:**
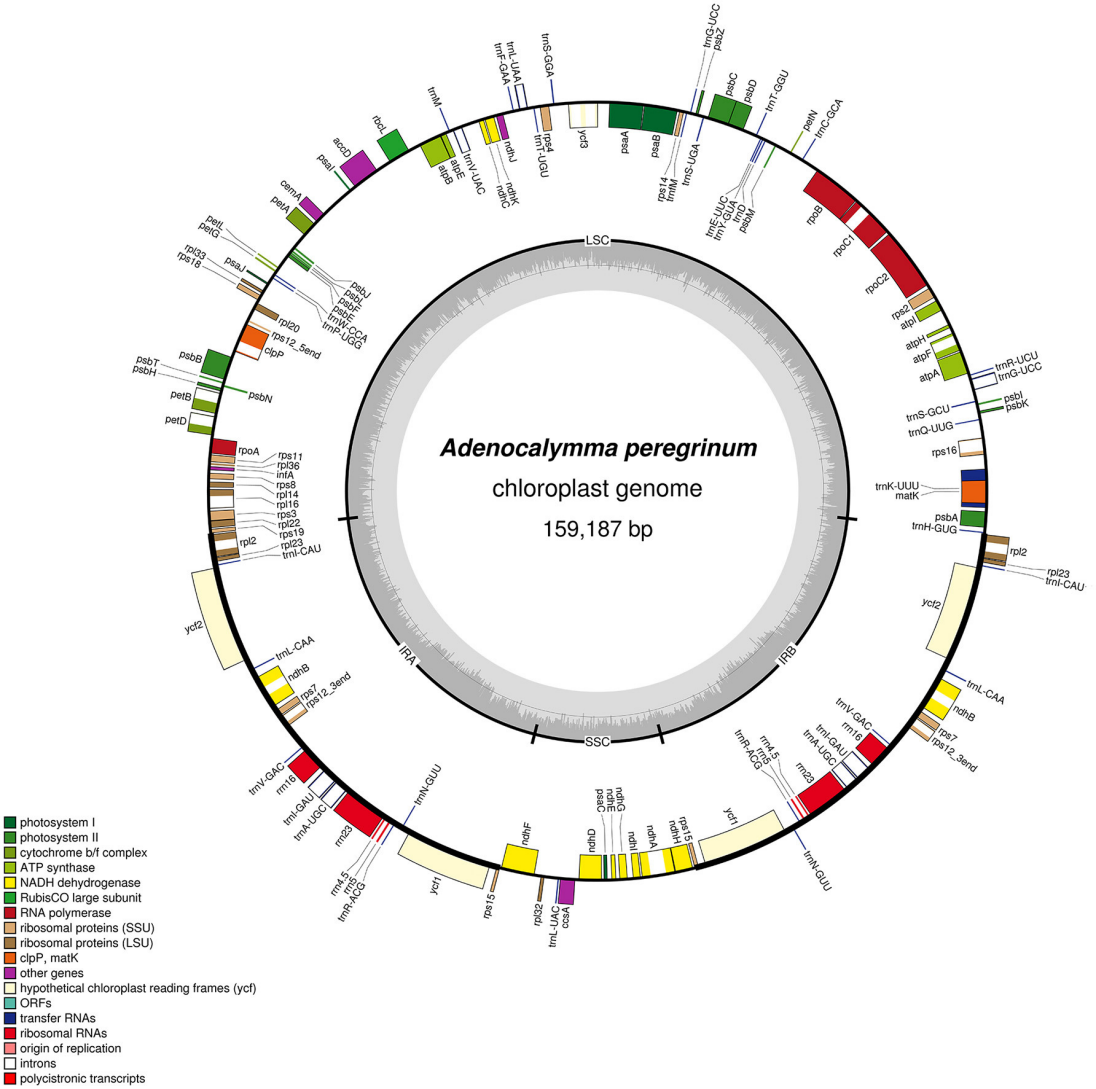
Gene map of the *A. peregrinum* chloroplast genome. Genes drawn inside the circle are transcribed clockwise, and those outside are transcribed counterclockwise. Genes belonging to different functional groups are color-coded. The darker gray in the inner circle corresponds to GC content, and the lighter gray corresponds to AT content.

**Figure 2:**
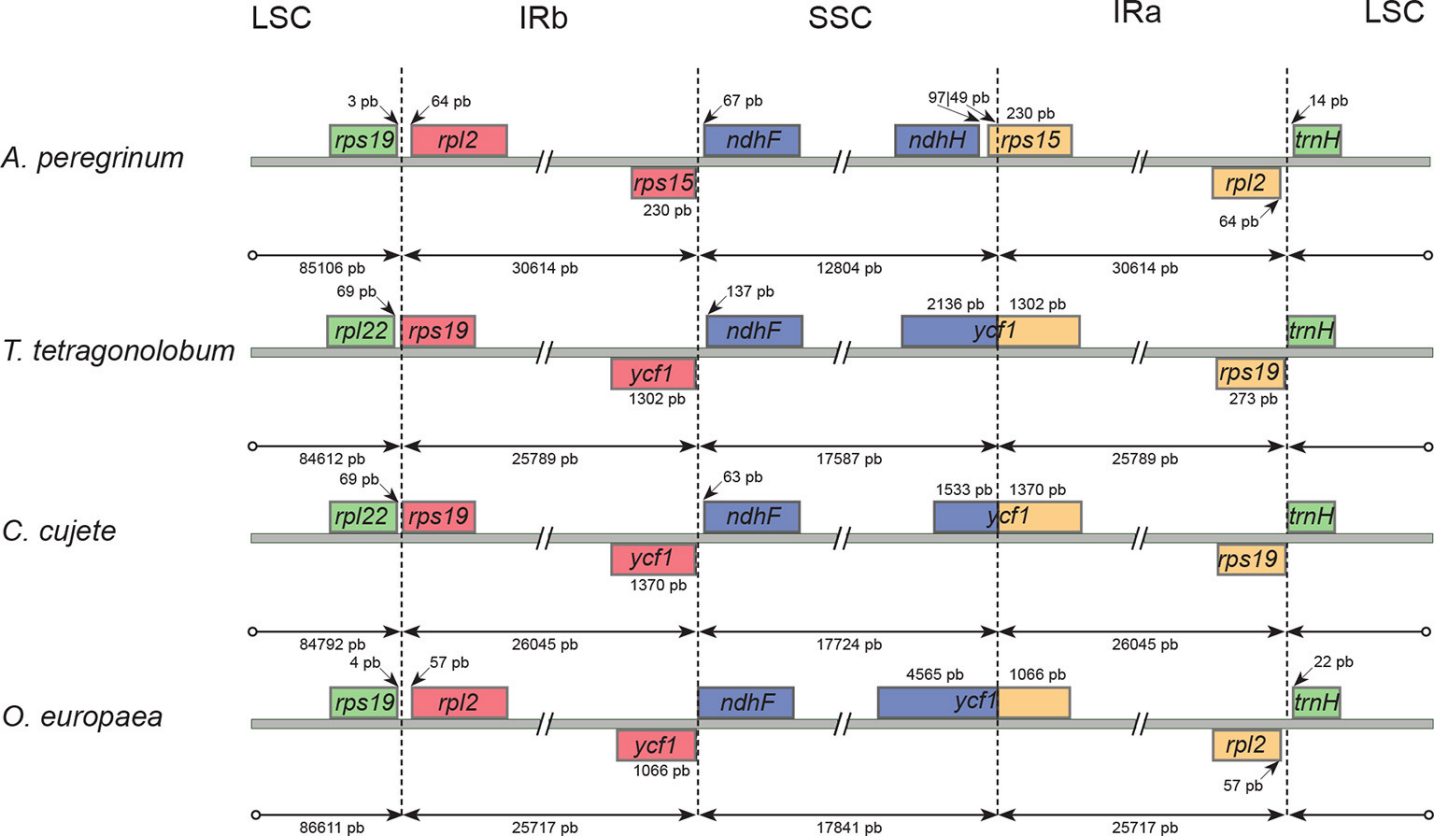
Comparisons of the Long Single Copy (LSC), Small Single Copy (SSC), and Inverted Repeated (IR) region borders among four Lamiales chloroplast genomes. Genes shown above the lines are transcribed forward while genes shown below the lines are transcribed reversely. Two-headed arrows indicate plastome partition sizes in base pairs and single-headed arrows indicate size of features or distances between plastome partition borders and features.

The plastomes of the 10 species of the “*Adenocalymma*-*Neojobertia*” clade ranged in size from 157,025 (*A. biternatum*) to 159,725 bp (*A. bracteatum*). All cp genomes have the typical quadripartite structure of Angiosperms, which consists of a pair of IR regions (30,084–30,954 bp) separated by a LSC region (84,059–85,665 bp), and a SSC region (12,585–12,804 bp). A circular map of *A. peregrinum* is shown (Figure 1), and those of all other species sampled are available as Supplemental Material (Supplementary Figures 1–9). The average GC content was ~38% for all species, with the exception of *A. nervosum* (41.6%). The GC content values suggest an AT-rich plastome organization, which is similar to that found in the other Bignoniaceae plastomes available to date (Nazareno et al., 2015; Moreira et al., 2016). All plastomes studied include 131–132 genes, with 86–87 coding regions, 37 tRNA, and 8 rRNA (Tables 2, 3). The difference in number of coding genes among species is due to the complete loss of the *ycf4* gene in *A. biternatum* and *A. peregrinum*. A copy of the duplicated gene *rps15* is a pseudogene, with only an incomplete sequence found at the border of the IRa and the SSC (Figure 2). Thirteen genes have one intron, while three genes (i.e., *clpP, rps12* and *ycf3*) have two introns (Table 3). In *rps12* a trans-splicing event was observed with the 5′ end located in the LSC region and the duplicated 3′ end in the IRs. The *trnQ-UUG* gene is duplicated in the LSC of all species, with one intron found in one of the copies (Table 3). Among protein-coding genes, 84–85 genes start with the standard initiator codon AUG; however, the *rps19* starts with GUG, while the *ndhD* starts with ACG. The stop codon UAA was the most common, followed by UAG and UGA.

**Table 3:** Genes recovered within the “*Adenocalymma-Neojobertia*” clade.

**Gene function**	**Gene type**	**Gene**
Self-replication	• rRNA genes • tRNA genes • Small ribosomal subunit • Large ribosomal subunit • DNA dependent RNA	*rrn4.5*^a^, rrn5^a^, rrn16^a^, rrn23^a^ trnA-UGC^a^^*^, trnC-GCA, trnD-GUC, trnE-UUC, trnF-GAA, trnfM-CAU, trnG-UCC, trnG-UCC^*^, trnH-GUG, trnI-CAU^*a*^, trnI-GAU^a^^*^, trnK-UUU^*^, trnL-CAA^a^, trnL-UAA^*^, trnL-UAG, trnM-CAU, trnN-GUU^a^, trnP-UGG, trnQ-UUG, trnR-ACG^a^ trnR-UCU, trnS-GCU, trnS-GGA, trnS-UGA, trnT-GGU, trnT-UGU, trnV-GAC^a^, trnV-UAC^*^, trnW-CCA, trnY-GUA *rps2, rps3, rps4, rps7*^a^, rps8, rps11, rps12^a^^**^, rps14, rps15 ^a^, rps16 ^*^, rps18, rps19 ^b^ rpl2^a^^*^, rpl14, rpl16^*^, rpl20, rpl22, rpl23^a^, rpl32, rpl33, rpl36 rpoA, rpoB, rpoC1^*^, rpoC2
Photosynthesis	• Photosystem I • Photosystem I • NADH-dehydrogenase • Cytochrome b6/f • complex • ATP synthase • Rubisco	psaA, psaB, psaC, psaI, psaJ, ycf3^**^ psbA, psbB, psbC, psbD, psbE, psbF, psbH, psbI, psbJ, psbK, psbL, psbM, psbN, psbT, psbZ ndhA^*^, ndhB^a^^*^, ndhC, ndhD, ndhE, ndhF, ndhG, ndhH, ndhI, ndhJ, ndhK petA, petB^*^, petD^*^, petG, petL, petN atpA, atpB, atpE, atpF^*^, atpH, atpI *rbcL*
Other genes	• Translational initiator • Maturase • Protease • Envelope membrane • protein • Acetil-CoA-carboxylase • c-type cytochrome • synthesis	*infA* *matK* clpP^**^ cemA *accD* *ccsA*
Unknown function	Conserved open read frames	*ycf1*^a^, ycf2^a^, ycf4^b^

* *Gene with one intron*.

** *Gene with two introns*.

a *Gene with two copies*.

b *Pseudogene in some species*.

The gene structure of the IR/SSC boundary regions were well conserved within the “*Adenocalymma-Neojobertia*” clade, only differing slightly in a few base pairs (Figure 2). However, major structural differences were observed when those plastomes were compared with those of *C. cujete, O. europaea*, and *T. tetragonolobum*. Species of the “*Adenocalymma-Neojobertia* clade” included the gene *ycf1* and part of the gene *rps15* at the borders of the IR and SSC regions; this led to a smaller SSC (12,585–12,804 bp) (Table 2) when compared to the SSC of *C. cujete* (17,724 bp), *O. europaea* (17,841 bp), and *T. tetragonolobum* (17,587 bp) (Figure 2). The IR regions and LSC borders found in members of the “*Adenocalymma-Neojobertia*” clade also differed from those of *C. cujete*, and *T. tetragonolobum*, with the *rps19* gene lacking from the IR regions of all species of the “*Adenocalymma-Neojobertia*” clade sampled (Figure 2). These rearrangements at the IR regions led to larger plastomes for all taxa analyzed (Table 2), when compared to those of *C. cujete* (154,662 bp), *O. europaea* (155,889 bp) and *T. tetragonolobum* (153,776 bp).

**Figure 3:**
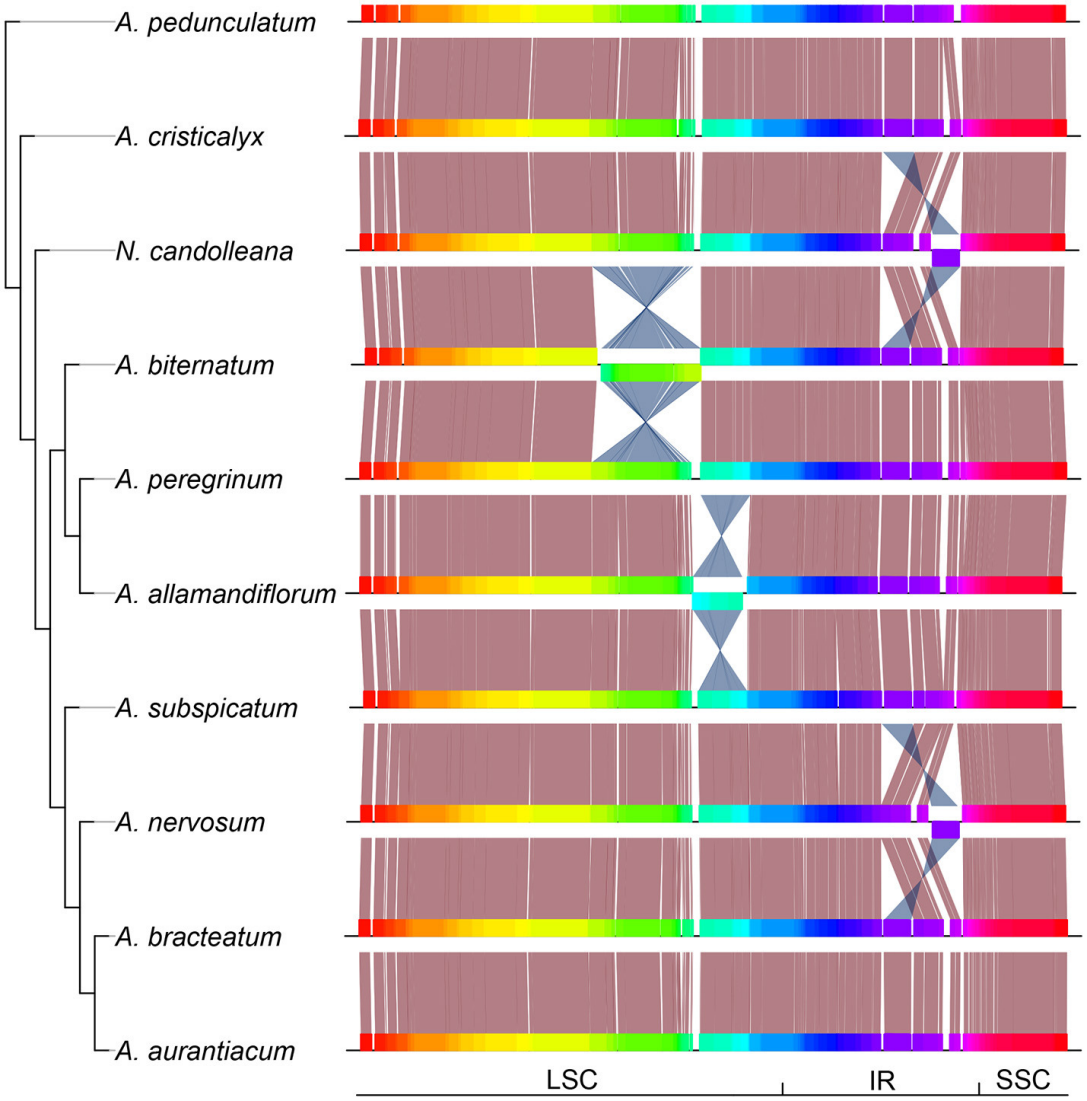
Phylogeny of the “*Adenocalymma-Neojobertia*” clade recovered from the analysis of the combined datasets from 10 representative species, followed by the linear plastid maps of all species sampled. Plastome regions are depicted with different colors; Salmon lines link conserved regions while blue lines link rearranged homologous regions. LSC, Long Single Copy region; SSC, Small Single Copy region; IR, Inverted Repeated region.

At least four major inversions were detected in some species of the “*Adenocalymma-Neojobertia*” clade. Two of those inversions were found at the LSC and two at the IRs (Figure 3). Rearrangements at the LSC occurred at different positions and were associated with different gene blocks (Figure 3). On the other hand, the rearrangements at the IRs involved the same gene blocks, except from the rearrangement found at *trnV-GAC*, indicating a parallel event. All structural changes involved non-coding regions. Furthermore, no genes were shut down by the inclusion of major genomic parts.

### Phylogenetic analyses

**Figure 4:**
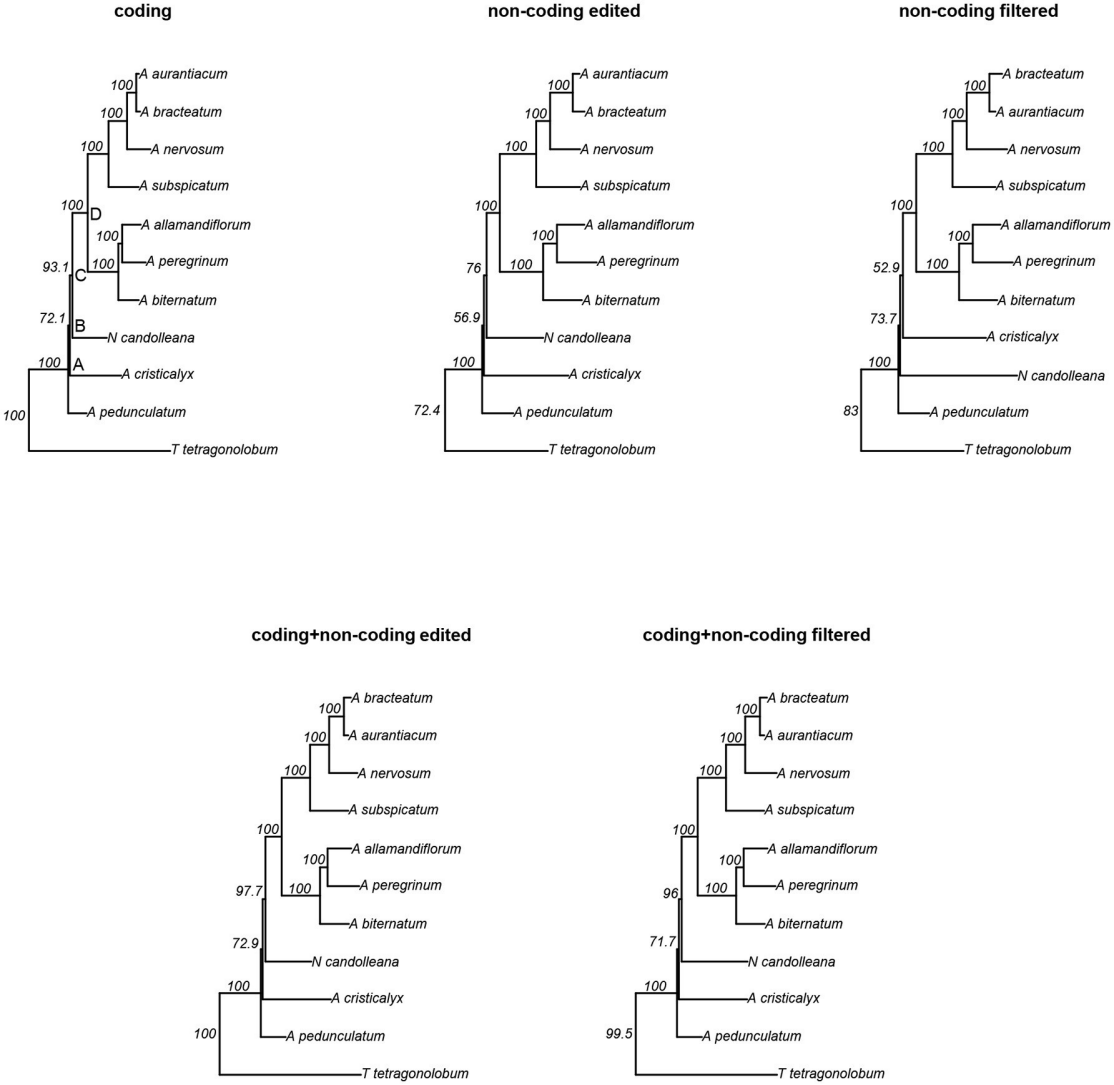
Maximum Likelihood (ML) trees derived from the analyses of five different partition schemes. Nodes A, B, C and D are depicted at the tree derived from the analyses of the “coding” region dataset. Values shown next to nodes are likelihood bootstrap support.

We conducted phylogenetic analyses of five different datasets derived from plastome data of 10 species belonging to the “*Adenocalymma-Neojobertia*” clade, plus three outgroups (i.e., *C. cujete, T. tetragonolobum*, and *O. europaea*) using ML and BC. Among all datasets, the “non-coding edited” and “non-coding filtered” datasets contained the highest number of variable sites (39.3%), followed by the “coding + non-coding edited” and “coding + non-coding filtered” datasets (32.5%), and the “coding” dataset (27.9%) (Table 4). The analyses of all datasets led to the same topology (Figure 4, Supplementary Figure 10), except from the topology reconstructed based on the “non-coding filtered” dataset, which led to a slightly different tree (Figure 4).

**Table 4:** Summary of partition schemes.

**Partition**	**Alignment length (bp)**	**Variable sites**	**% of variation**
Coding	71,395	19,912	27.9
Non-coding edited	48,469	19,052	39.3
Non-coding filtered	48,319	19,005	39.3
Coding + non-coding edited	119,864	38,964	32.5
Coding + non-coding filtered	119,714	38,917	32.5

All topologies derived from the BC and ML analyses recovered *A. pedunculatum* as sister to all other species of the “*Adenocalymma-Neojobertia* clade” (node A). For the majority of the topologies, node A is followed by the divergence of *A. cristicalyx* (node B), which is followed by the divergence of *N. candolleana* (node C). The remaining species are included in a clade (node D) that is divided into two sub-clades, one including (*A. biternatum, A. allamandiflorum, A. peregrinum*) and the other including (*A. subspicatum, A. nervosum, A. aurantiacum, A. bracteatum*) (Figure 4, Supplementary Figure 10). Node D and all clades included herein were recovered from the analyses of all datasets. However, nodes B and C were not recovered in the tree that resulted from the analyses of the “non-coding filtered” dataset for both BC and ML; instead, the analyses of the “non-coding filtered” dataset recovered *N. candolleana* as the second diverging lineage (right after node A) within the “*Adenocalymma-Neojobertia*” clade. This node is followed by the divergence of *A. cristicalyx* (Figure 4, Supplementary Figure 10).

Bootstrap mean values of trees derived from the analyses of the datasets are: 96.3% for the “coding” dataset, 92.54% for the “non-coding edited” dataset, 91.85% for the “non-coding filtered” dataset, 96.73% for the “coding + non-coding edited” dataset, and 96.41% “coding + non-coding filtered” dataset. Most nodes have maximum support in all trees, except from nodes B and C, where all differences in support are found. Among the combined datasets, the tree derived from the analysis of the “coding + non-coding edited” dataset has bootstrap values of 72.9 and 97.7 for nodes B and C, respectively and the “coding + non-coding filtered” dataset has bootstrap values of 71.7 and 96 for nodes B and C, respectively (Figure 4).

### Identification of markers for species level phylogenetic studies

The five (out of 31) regions with highest potential for species level phylogenetic studies based on the percentage of sequence variation, topological and branch length distances were: *clpP* intron 1, *ndhA* intron, *petN-psbM* spacer, *rpl32-trnL* spacer, and *trnG* intron (Table 5, Supplementary Table 2). Three out of the five regions selected are part of the LSC; with *ndhA* intron and *rpl32-trnL* spacer included in the SSC. The *ndhA* intron was the region with the greatest percentage of sequence variation, followed by the *petN-psbM* spacer, *trnG* intron, *clpP* intron 1, and *rpl32-trnL* spacer (Table 5). The topologies obtained from the analysis of the *petN-psbM*, and *trnG* intron identical to the best plastome tree (i.e., the tree derived from the analysis of the “coding + non-coding edited” dataset). Among the five regions selected, the trees derived from the analyses of the *clpP* intron 1, and *trnG intron* spacer datasets were the most similar to the best plastome tree in terms of branch lengths (Table 5). Primers for PCR amplification were designed for four regions selected (Table 6).

**Table 5:** Summary statistics of the five most useful introns and intergenic spacers for phylogeny reconstruction.

**Region**	**Alignment length (bp)**	**Min. seq. length (bp)**	**Max. seq. length (bp)**	% of variation^*^	Topology dist.^*^^a^	Branch lengths dist.*^a^
*ndhA* intron	1173	958	1109	1	0.85	0.89
*petN-psbM*	1233	986	994	0.39	1	0.79
*trnG* intron	706	670	699	0.2	1	0.91
*clpP* intron 1	852	732	750	0.21	0.89	0.9
*rpl32-trnL*	961	863	889	0.64	0.59	0.75

Regions were selected based on a standardized mean of three variables:

(1) Percentage of variable sites;

(2) Phylogenetic tree topology distance and

*(3) Phylogenetic tree branch lengths distance*.

* *Values standardized*.

a *Distances computed using Kendall and Colijn (2016) method*.

**Table 6:** Sequences of primer pairs designed in this study for selected regions.

**Region**	**Forward primer**	**Reverse primer**	**Tm (°C)**
*ndhA* intron	TCAATATCTCTACGTGCGATTCG	CCGTCGCTATTACAGAACCGT	59.5
*petN–psbM*	TGGGGAAGAAGTGGGCTCTA	AGTTCCTACCGCTTTTCTACTT	59.9
*trnG* intron	TGTAGCGGGTATAGTTTAGTGGT	CGCATCGTTAGCTTGGAAGG	59
*clpP* intron 1	AGGACCGTACATGCACCTTT	CCTCTGTTTCGCCCAAGAAA	58.9

## Discussion

In this study, we sequenced, assembled and annotated the plastomes of nine species of *Adenocalymma* and the plastome of *Neojobertia candolleana*. The assembled plastomes were compared with those from *C. cujete, O. europaea*, and *T. tetragonolobum*. Phylogenetic studies using five data partition schemes were conducted and compared in terms of topology and bootstrap support. Overall, the “coding + non-coding edited” dataset led to the best estimate of phylogenetic relationships within the “*Adenocalymma-Neojobertia*” clade, representing the best dataset for phylogenetic studies. A search for variable regions for phylogenetic studies identified the five markers with the highest potential for species level phylogenetic studies. Primers were designed for four regions and are now available for future phylogenetic studies within the “*Adenocalymma-Neojobertia*” clade and the Bignoniaceae as a whole. These results establish a foundation for future studies on the evolution of plastome structure and phylogenomics within the Bignoniaceae.

### Plastome features

Seed plant plastomes typically encode up to 80 protein coding genes, 30 tRNAs and eight rRNAs (Wu and Chaw, 2015; Asaf et al., 2016; Reginato et al., 2016). Differences in plastome size are usually a result of IR expansions or contractions (Kim and Lee, 2004). Plastome architecture is highly conserved in Seed Plants (Odintsova and Yurina, 2003; Wicke et al., 2011; Smith and Keeling, 2015; Wu and Chaw, 2015; Reginato et al., 2016), with only a few examples of plastic genome architecture available for Angiosperms (e.g., Guisinger et al., 2011) and Gymnosperms (e.g., Wu and Chaw, 2016). The plastomes of selected members of the “*Adenocalymma-Neojobertia*” clade include similar numbers of genes than previously sequenced plastomes (Hu et al., 2015). More specifically, the plastomes of members of the “*Adenocalymma-Neojobertia*” clade include 86–87 protein coding genes, 37 tRNAs and eight rRNAs (Table 2). However, when the newly sequenced plastomes are compared with those from other Bignoniaceae (i.e., *C. cujete* and *T. tetragonolobum*), a pronounced expansion of the IRs and a contraction of the SSC were encountered, with the complete inclusion of the gene *ycf1* and part of the *rps15* in the IRs (Figure 2). Although unusual, the expansion of the IRs toward the SSC has also been reported in *Pelargonium* L'Hér. (Chumley et al., 2006) and members of Apiales (Downie and Jansen, 2015). Furthermore, a pseudogene was found in the plastomes of all species of the “*Adenocalymma-Neojobertia*” clade, with the partial loss of *rps15* from the IR of all species sampled, and complete loss of *ycf4* from the LSC in *A. biternatum* and *A. peregrinum* (Table 3). Pseudogenization events (gene duplication followed by loss of function) have been reported in several plant lineages. A notable example is the transfer of the *accD* gene from the plastid to the nucleous of *Primula sinensis* Sabine ex Lindley (Liu et al., 2016). Pseudogenes are also common at the IRa/IRb and LSC junction regions, with loss of function due to the accumulation of premature stop codons or gene loss, which is particularly common for *ycf1* and *rps19* (Nazareno et al., 2015; Moreira et al., 2016).

The structure of the whole plastome was also found to be quite variable, with rearrangements in the LSC and IRs regions (Figure 3). This plastic architecture has also been reported for the Geraniaceae (Guisinger et al., 2011) and Mimosoid Legumes (Dugas et al., 2015). Genic regions are usually conserved, with rearrangements occurring predominantly at intergenic regions (Dugas et al., 2015). Furthermore, several genes are transcribed in operons due to the endosymbiotic origin of plastomes (Sugita and Sugiura, 1996; Sugiura et al., 1998). These gene clusters are stretches of the plastome consisting of several genes (Sugita and Sugiura, 1996; Sugiura et al., 1998), explaining the relative conserved pattern of gene groups and the frequent rearrangements that are found in spacers between gene clusters (Dugas et al., 2015).

### Phylogenetic analyses

Plastome sequences have been successfully used to address phylogenetic questions at different taxonomic scales using both protein coding and non-coding sequences (e.g., Soltis et al., 1999; Shaw et al., 2007). Here, we used plastome sequences of ten species of the “*Adencalymma-Neojobertia*” clade and three outgroups (i.e., *C. cujete, O. europaea* and *T. tetragonolobum*) to reconstruct major phylogenetic affinities within this clade. We also compared five different data partition schemes in order to determine the best dataset for phylogenetic studies. The most variable regions were the introns and spacers (39.3%), with protein coding regions showing a much lower number of informative sites (27.9%) (Table 4). Higher rates of molecular evolution in intronic and intergeneic regions have also been reported for several other plant groups (e.g., *Begonia* L., Harrison et al., 2015; *Epimedium*, Zhang et al., 2016; Melastomataceae, Reginato et al., 2016). There is growing evidence that organellar genomes, including plastomes, are not a direct product of natural selection, but may have been shaped by adaptative and non-adaptive processes (Lynch et al., 2006; Lynch, 2007). As a result, non-coding regions may be more prone to indel events and a higher number of DNA substitutions when compared to coding regions.

Phylogenies were estimated using five data partitions independently. The topologies recovered using ML and BC are highly concordant, regardless of the dataset used (Figure 4, Supplementary Figure 10). The “non-coding filtered” dataset was the only data partition that led to a different topology when compared with other datasets in both criteria (Figure 4, Supplementary Figure 10). For this dataset poorly aligned regions and indels were removed using Gblocks and outlier sequences were removed using T-Coffee. However, even after a pure mechanistic approach non-homologous portions derived from rearrangements remained aligned, leading to the difference in topologies observed. Indeed, rearrangements can lead to a loss of homology correspondence in particular genomic regions which, when aligned, increase the number of gaps and “saturated” regions in sequence alignments (Castresana, 2000; Xia et al., 2003; Jeffroy et al., 2006; Misof et al., 2014). Indels and saturated regions are putatively eliminated with Gblocks (Castresana, 2000), but with some limitations to deal with rare misaligned sequences. T-Coffee was used the remove the sequences (Notredame et al., 2000), however even using different thresholds of sequence similarity some outliers remained, leading to a different topology when compared to “coding” and “non-coding edited” datasets (Figure 4, Supplementary Figure 10).

The analyses of all combined datasets (i.e., “coding + non-coding edited,” and “coding + non-coding filtered”) recovered identical topologies and similar branch lengths in all BC and ML searches (Figure 4, Supplementary Figure 10), thus revealing the importance of the phylogenetic signal of the coding regions (Figure 4, Supplementary Figure 10). However, a small increase in bootstrap support at nodes B and C is observed in the tree that resulted from the analysis of the “coding + non-coding edited” dataset (Figure 4), suggesting a decrease of phylogenetic noise in the dataset with non-homologous sequences derived from rearrangements removed by hand (Figure 4, Supplementary Figure 10) when compared with the dataset computationally edited. Overall, our results suggest that the “coding + non-coding edited” dataset is the most reliable data partition for phylogenetic estimation within the “*Adenocalymma-Neojobertia*” clade due to the greater node support (Jeffroy et al., 2006; Misof et al., 2014). In the case of inclusion of non-coding regions, alignment visual inspection is necessary to prevent non-homologous regions prevenient from rearrangements being included after constructing the datasets by sequence similarity.

### Identification of markers for species level phylogenetic studies

The genomic data obtained in this study allowed us to identify the four most promising plastome regions for phylogeny reconstruction within the “*Adenocalymma-Neojobertia*” clade. Despite the limited sampling (approximately 15% of the known species), the sampled taxa cover the breath of morphological diversity found within the “*Adenocalymma-Neojobertia*” clade and are broadly distributed through the phylogeny of this clade (Fonseca and Lohmann, in prep.). Therefore, the regions selected likely represent good markers for phylogeny reconstruction within the whole clade. Among the regions selected, the *ndhA* intron also showed a high potential for phylogeny reconstruction in the Melastomataceae (Reginato et al., 2016), *rpl32-trnL* is an intergenic region widely used among angiosperms (Shaw et al., 2007). The *rpl32-trnL* marker has been successfully used in phylogenetic studies within the Bignoniaceae (Fonseca and Lohmann, 2015; Medeiros and Lohmann, 2015). While high-throughput sequencing methods allow the generation of an enormous amount of data, budget and computational limitations can reduce the taxonomic coverage of studies of this nature. To ease some of these limitations, a hybrid NGS and Sanger sequencing approach is recommended and has been successfully used to reconstruct the phylogeny of a variety of plant lineages, including the Malpighiales (Xi et al., 2012), Arundinarieae-Poaceae (Ma et al., 2014), and Goodeniaceae (Gardner et al., 2016). Indeed, a combination of NGS and Sanger data may represent the most cost-efficient approach to estimate species-level phylogenies.

## Conclusions and future directions

Ten full plastomes of species from the “*Adenocalymma-Neojobertia*” clade led to a strongly supported phylogeny for this lineage. The plastomes assembled also allowed the identification of four suitable molecular markers for future phylogenetic studies. The plastic nature of the genomic architecture of members of this clade has direct implications for plastome assembly. More specifically, the recurrent rearrangements indicate the importance of *de novo* strategies for plastome assembly. Given that rearrangements occur even between closely related species, reference based approaches are not advisable. This variable architecture also has implications for phylogenomics as the lack of correspondence between gene junctions leads to problematic sequence alignments and errors in sequence homology assessment. The resulting bias can be reduced by the exclusion of poorly aligned regions.

The results derived from this study also serve as basis for future phylogenetic work within the “*Adenocalymma-Neojobertia*” clade. Ongoing studies, based on a broader sampling of taxa (approximately 90% of the known species of the “*Adenocalymma-Neojobertia*” clade) and a combination of Sanger and NGS sequencing data, aim to reconstruct a comprehensive phylogeny for the whole clade (Fonseca and Lohmann, in prep.). A robust phylogeny of this taxonomically complicated group, based a broad sample of taxa and markers, is critical to evaluate the monophyly of taxa, identify potential morphological synapomorphies for lineages, and subsidize taxonomic studies in this group (Fonseca and Lohmann, in prep.).

Our results also have major implications for broader phylogenetic studies within the whole Bignoniaceae. More specifically, the four molecular markers identified as suitable for phylogenetic studies within the “*Adenocalymma-Neojobertia*” clade, could also be used to reconstruct phylogenetic relationships within the whole family. A broad phylogeny is already available for the Bignoniaceae (Olmstead et al., 2009), however support of deeper relationships could be substantially improved by an increase in sampling of taxa and markers. A robust phylogeny of the whole Bignoniaceae is critical for an improved understanding of the biogeographic and evolutionary history of this ecologically diverse clade of Neotropical trees, shrubs and lianas (Gentry, 1980).

## Author contributions

LF and LL conceived and designed the experiment, collected the materials, and wrote the paper. LF performed the experiments, assembled sequences, and analyzed the data.

### Conflict of interest statement

The authors declare that the research was conducted in the absence of any commercial or financial relationships that could be construed as a potential conflict of interest.
