# Supplementary material for: Plastome Rearrangements in the “Adenocalymma-Neojobertia” Clade (Bignonieae, Bignoniaceae) and Its Phylogenetic Implications
Source: Front Plant Sci. 2017 Nov 1;8:1875. doi: 10.3389/fpls.2017.01875 (PMC5672021; doi:10.3389/fpls.2017.01875)
Supplement: Supplementary file 1 [file Supplementarymaterial.docx]

Supplementary Material

Plastome rearrangements in the “*Adenocalymma-Neojobertia*” clade (Bignonieae, Bignoniaceae) and its phylogenetic implications

Luiz Henrique M. Fonseca^1,2^, Lúcia G. Lohmann^1,3^

*** Correspondence:** LHMF: [luizhmf@gmail.com^2^](mailto:luizhmf@gmail.com2); LGL: [llohmann@usp.br](mailto:llohmann@usp.br)^3^

# Supplementary Figures and Tables

## Supplementary Figures

## Supplementary Tables


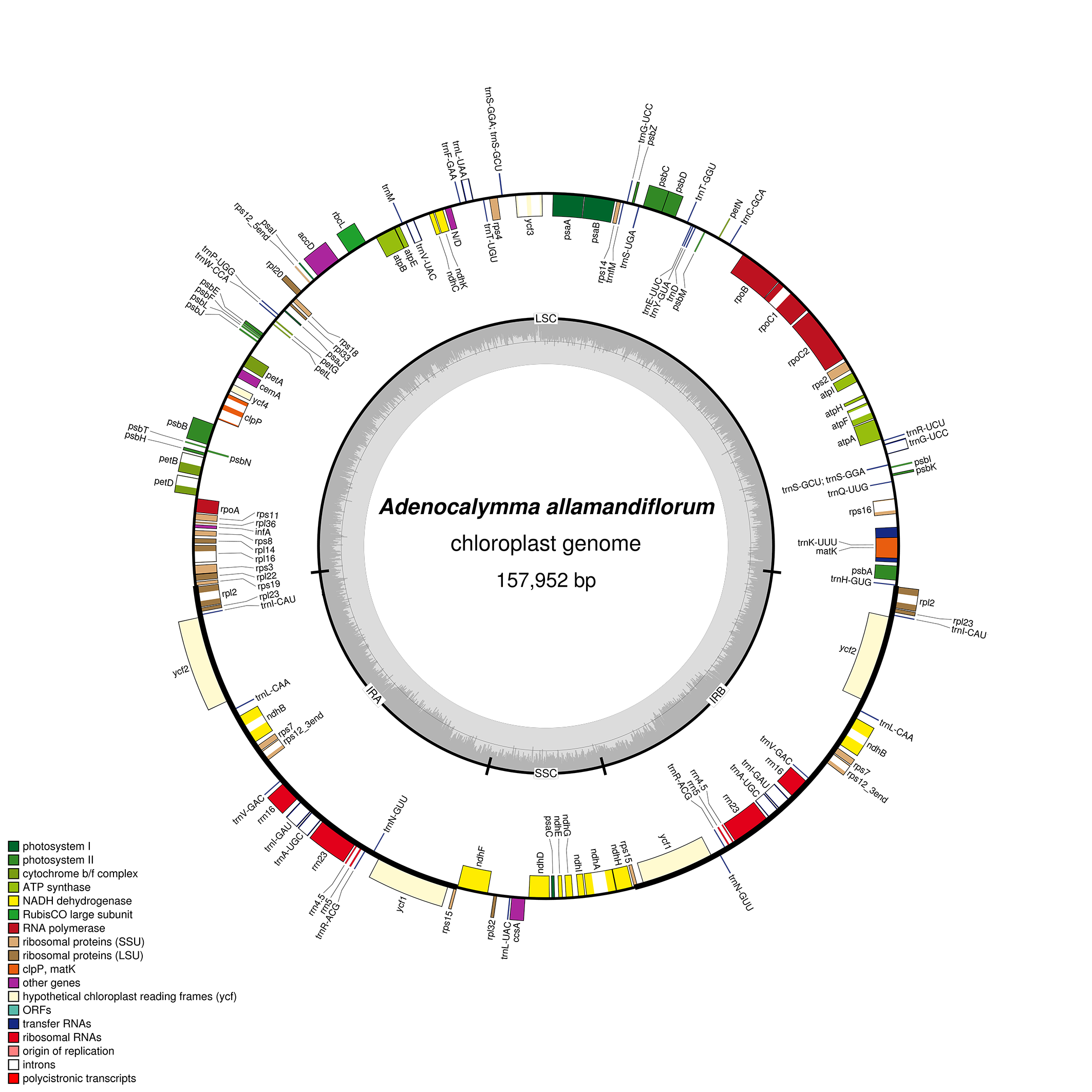


**Supplementary Figure 1.** Gene map of the *Adenocalymma alamandiflorum* chloroplast genome. Genes drawn inside the circle are transcribed clockwise, and those outside are counterclockwise. Genes belonging to different functional groups are color-coded. The darker gray in the inner circle corresponds to GC content, and the lighter gray corresponds to AT content.


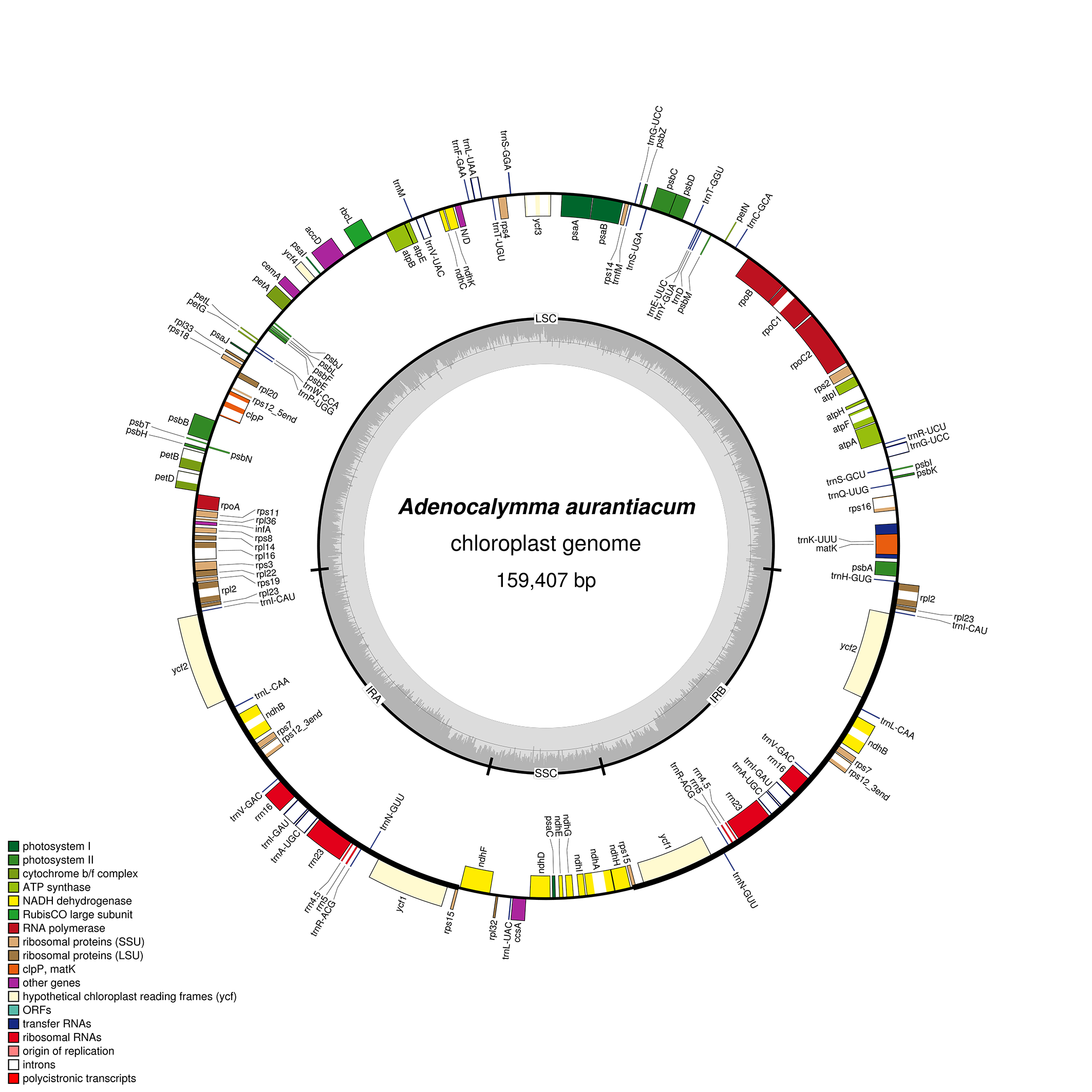


**Supplementary Figure 2.** Gene map of the *Adenocalymma auranticaum* chloroplast genome. Genes drawn inside the circle are transcribed clockwise, and those outside are counterclockwise. Genes belonging to different functional groups are color-coded. The darker gray in the inner circle corresponds to GC content, and the lighter gray corresponds to AT content.


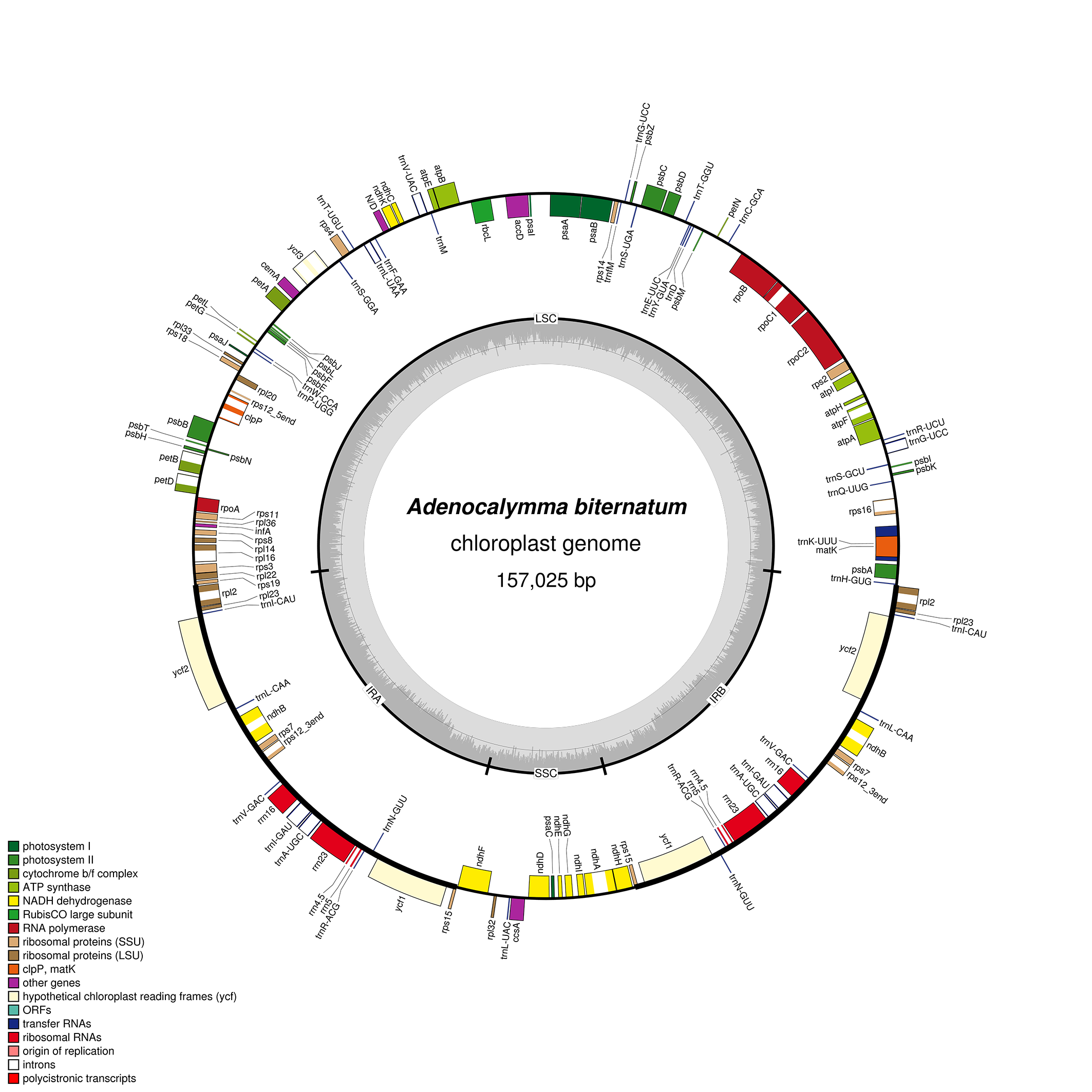


**Supplementary Figure 3.** Gene map of the *Adenocalymma biternatum* chloroplast genome. Genes drawn inside the circle are transcribed clockwise, and those outside are counterclockwise. Genes belonging to different functional groups are color-coded. The darker gray in the inner circle corresponds to GC content, and the lighter gray corresponds to AT content.


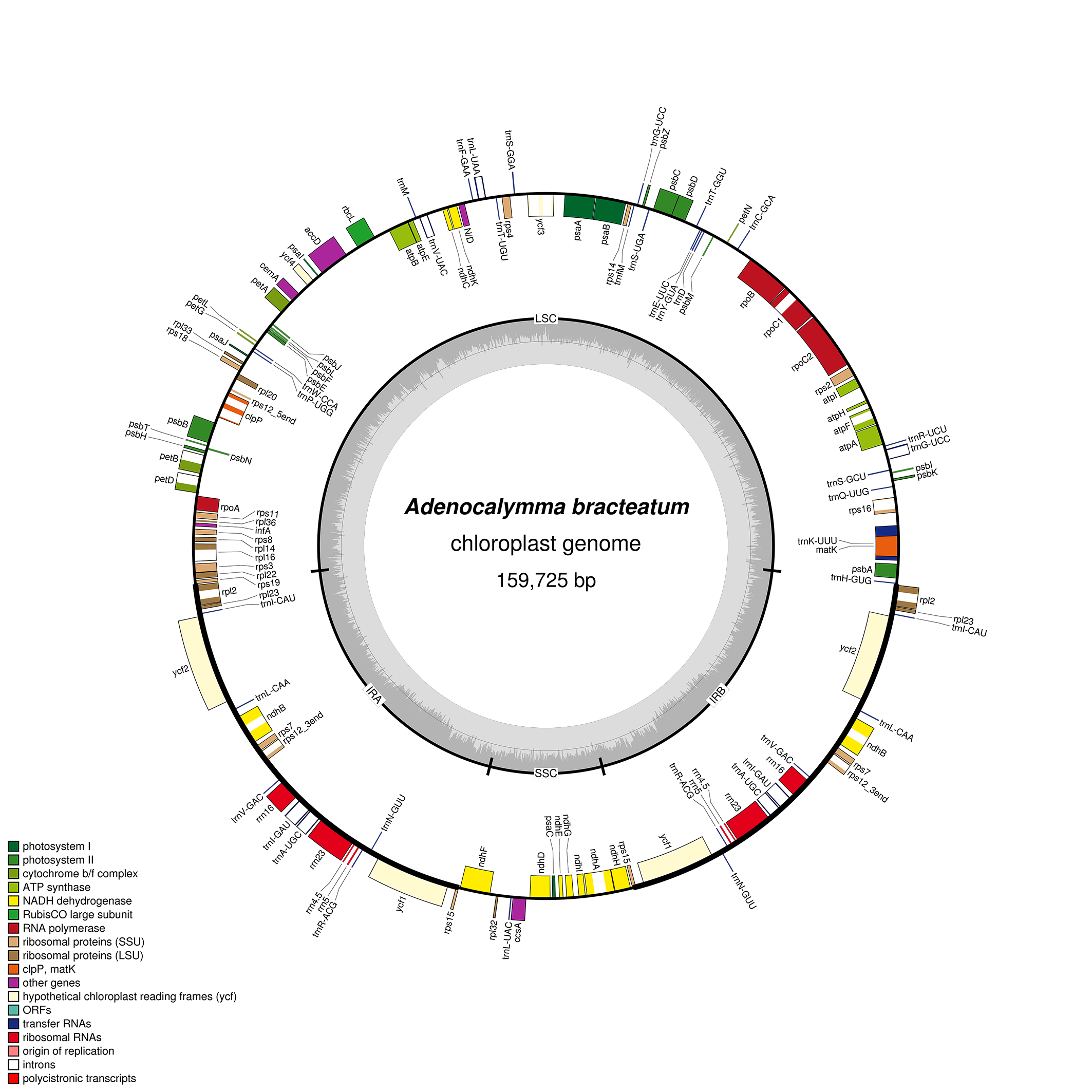


**Supplementary Figure 4.** Gene map of the *Adenocalymma bracteatum* chloroplast genome. Genes drawn inside the circle are transcribed clockwise, and those outside are counterclockwise. Genes belonging to different functional groups are color-coded. The darker gray in the inner circle corresponds to GC content, and the lighter gray corresponds to AT content.


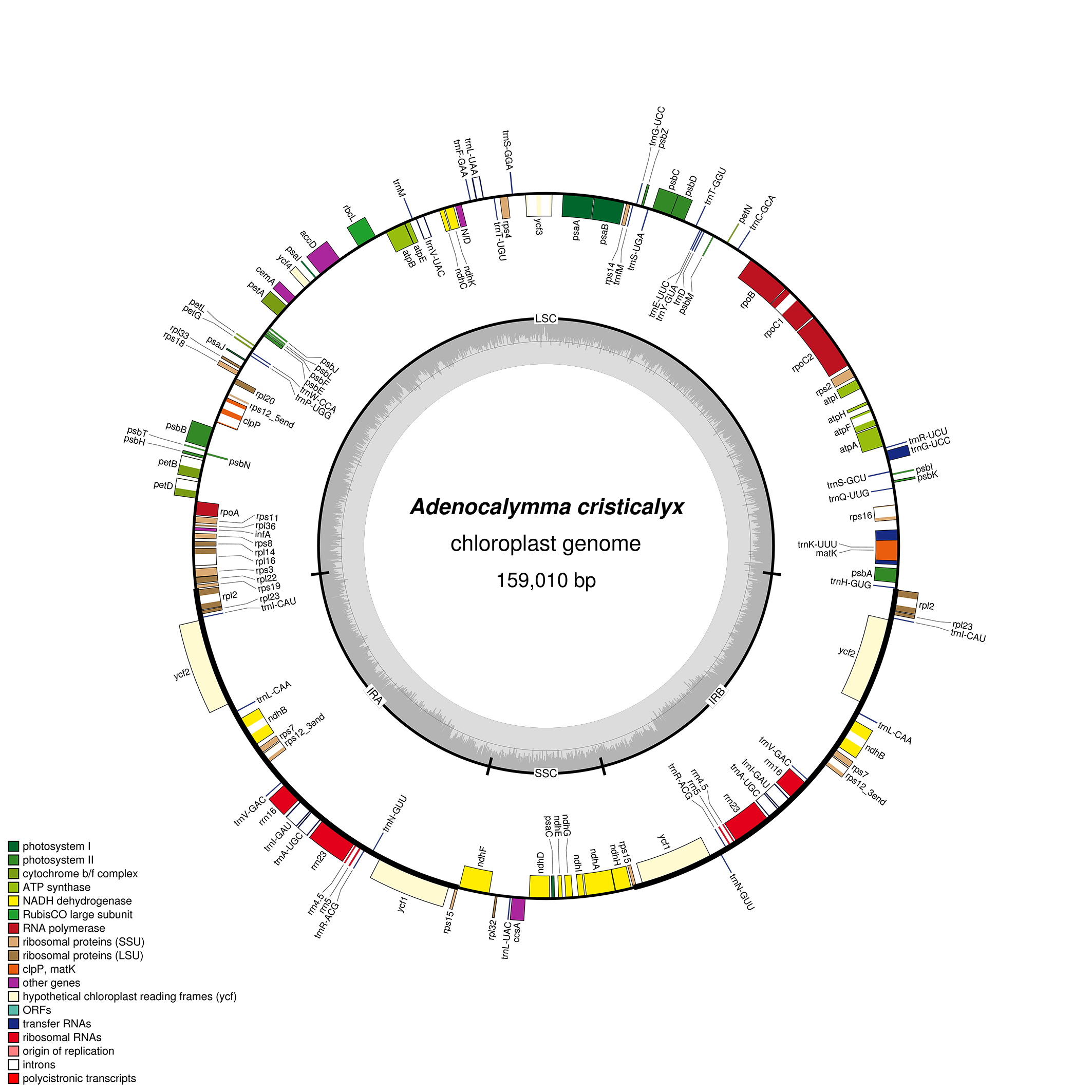


**Supplementary Figure 5.** Gene map of the *Adenoalymma cristicalyx* chloroplast genome. Genes drawn inside the circle are transcribed clockwise, and those outside are counterclockwise. Genes belonging to different functional groups are color-coded. The darker gray in the inner circle corresponds to GC content, and the lighter gray corresponds to AT content.


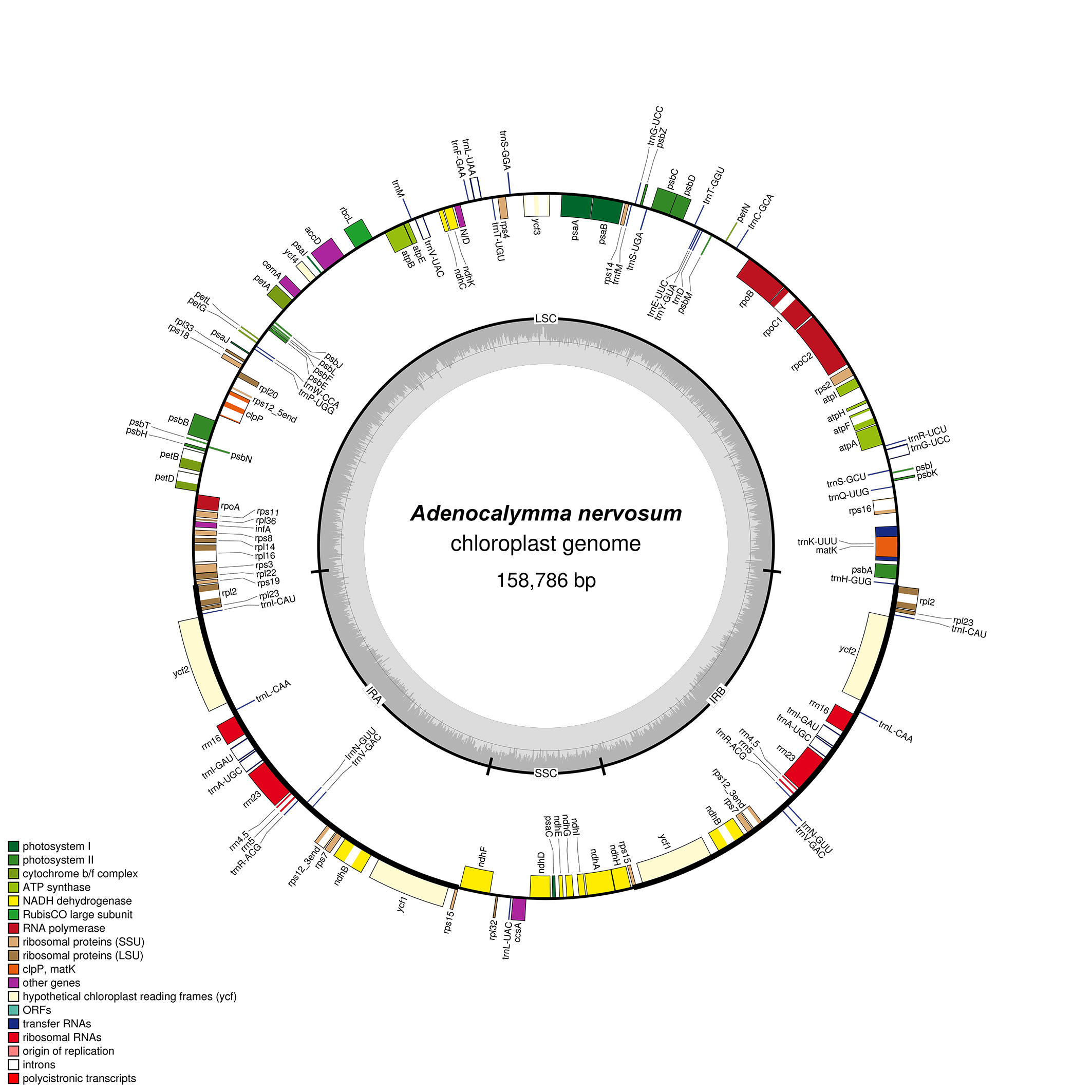


**Supplementary Figure 6.** Gene map of the *Adenocalymma nervosum* chloroplast genome. Genes drawn inside the circle are transcribed clockwise, and those outside are counterclockwise. Genes belonging to different functional groups are color-coded. The darker gray in the inner circle corresponds to GC content, and the lighter gray corresponds to AT content.


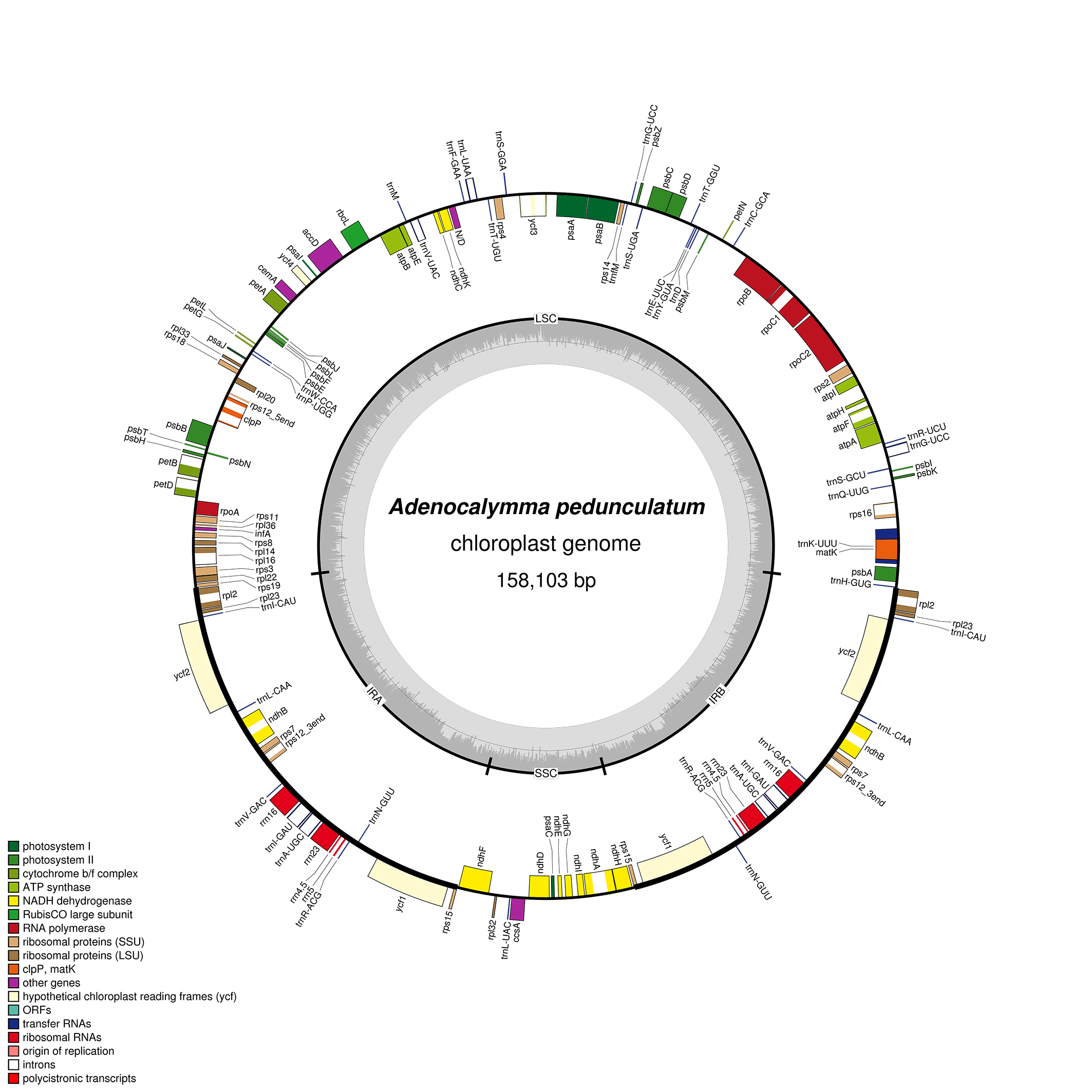


**Supplementary Figure 7.** Gene map of the *Adenocalymma pedunculatum* chloroplast genome. Genes drawn inside the circle are transcribed clockwise, and those outside are counterclockwise. Genes belonging to different functional groups are color-coded. The darker gray in the inner circle corresponds to GC content, and the lighter gray corresponds to AT content.


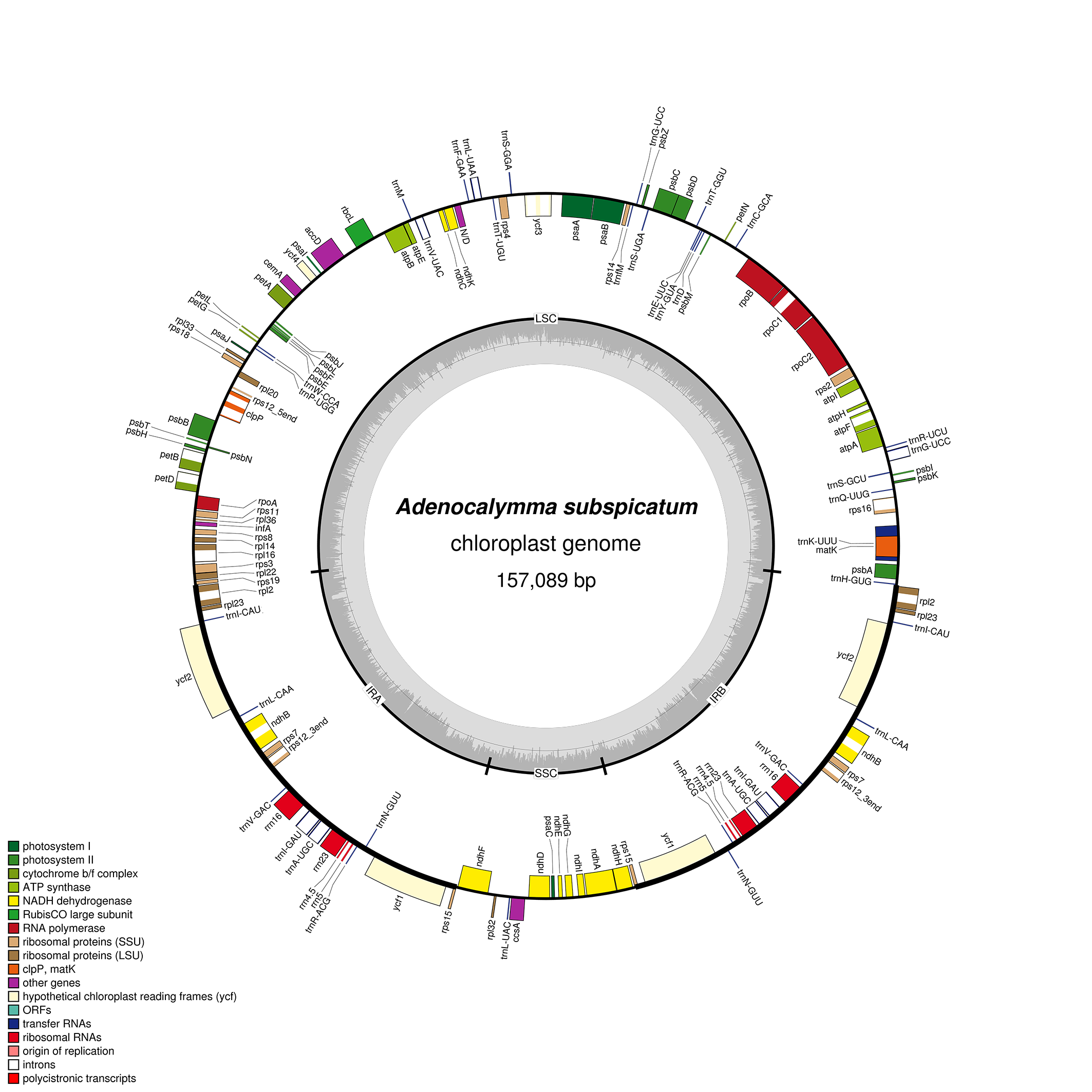


**Supplementary Figure 8.** Gene map of the *Adenocalymma subspicatum* chloroplast genome. Genes drawn inside the circle are transcribed clockwise, and those outside are counterclockwise. Genes belonging to different functional groups are color-coded. The darker gray in the inner circle corresponds to GC content, and the lighter gray corresponds to AT content.


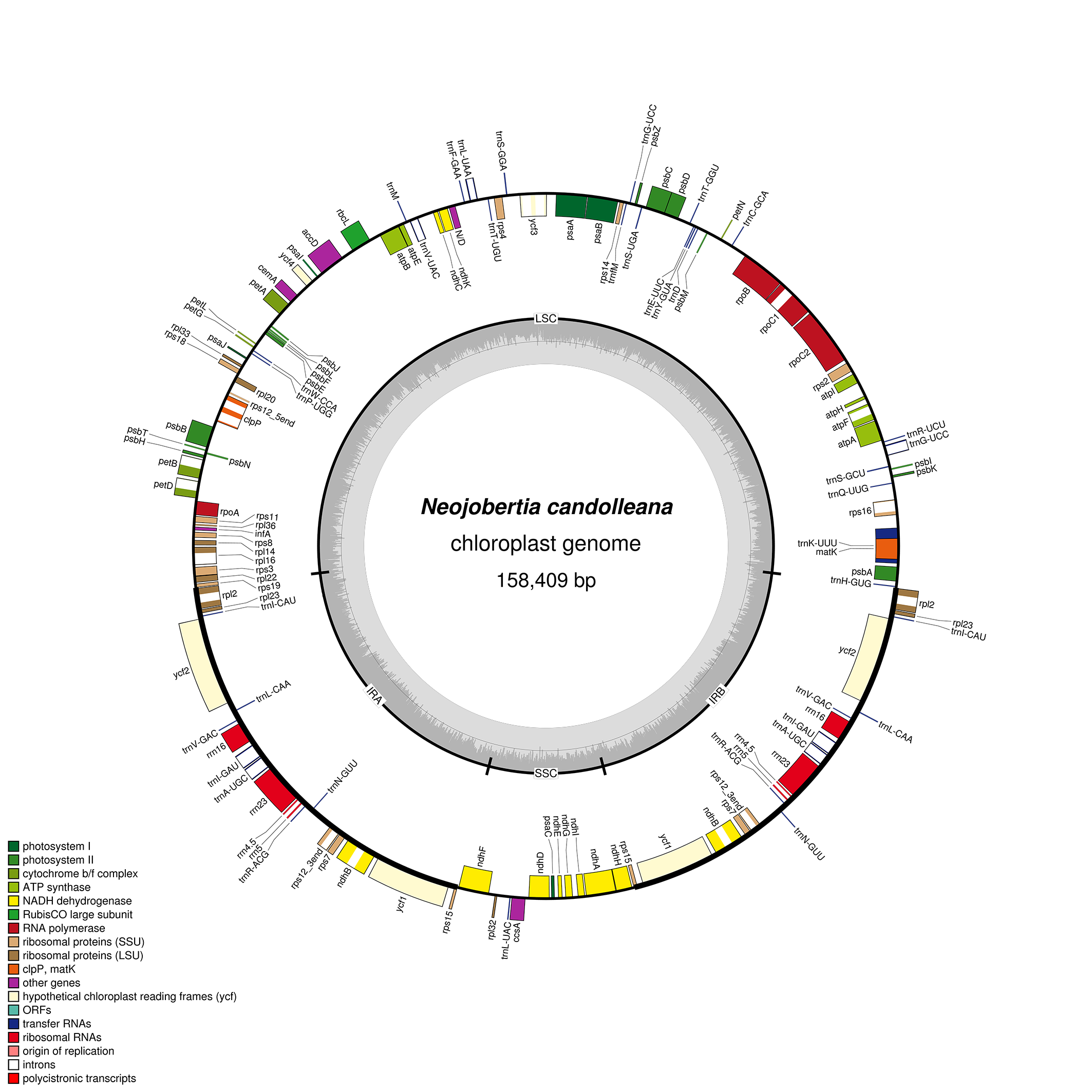


**Supplementary Figure 9.** Gene map of the *Neojobertia candolleana* chloroplast genome. Genes drawn inside the circle are transcribed clockwise, and those outside are counterclockwise. Genes belonging to different functional groups are color-coded. The darker gray in the inner circle corresponds to GC content, and the lighter gray corresponds to AT content.

**Supplementary Figure 10.** Trees derived from the Bayesian analyses of six different partition schemes. Values next to nodes are posterior probabilities.

## 1.2. Supplementary Tables

**Supplementary Table 1.** Mean coverage of whole partitions and intergenic regions among species where rearrangements are inferred.

| **Species** | **Partition** | **Partition mean coverage (x)** | **Inversion point 1 coverage (x)** | **Inversion point 2 coverage (x)** | **Inversion point 3 coverage (x)** |
| --- | --- | --- | --- | --- | --- |
| *Adenocalymma allamandiflorum* | LSC | 205 | 183.5 | 190 | – |
| *Adenocalymma biternatum* | LSC | 370.5 | 290.5 | 160.5 | – |
| *Adenocalymma nervosum* | IR | 682.8 | 908.6 | 766.4 | 800.9 |
| *Neojobertia candolleana* | IR | 1665.6 | 1008.8 | 1468 | 855.9 |

**Supplementary Table 2.** Summary statistics of introns and intergenic spacers selected. In bold, the five best regions ranked based on a standardized mean of three variables; (1) percentage of variable sites; (2) phylogenetic tree topology distance, (3) and phylogenetic tree branch length distance. Raw values of topological and branch length distances are provided.

| **Region** | **Alignment length (bp)** | **Min. seq. length (bp)** | **Max. seq. length (bp)** | **% variable sites** | **Topology dist.** | **Branch length dist.** |
| --- | --- | --- | --- | --- | --- | --- |
| ***ndhA intron*** | **1173** | **958** | **1109** | **0.214** | **2** | **0.017748** |
| ***psbM–petN*** | **1233** | **986** | **994** | **0.094** | **0** | **0.033787** |
| ***trnG intron*** | **706** | **670** | **699** | **0.07** | **0** | **0.014532** |
| ***clpP intron 1*** | **852** | **732** | **750** | **0.069** | **1.414213** | **0.015696** |
| ***rpl32–trnL*** | **961** | **863** | **889** | **0.173** | **5.385164** | **0.040944** |
| *trnV–ndhC* | 1207 | 1120 | 1140 | 0.092 | 5.385164 | 0.022610 |
| *petA–psbJ* | 1106 | 985 | 1019 | 0.082 | 5.744562 | 0.012759 |
| *psbE–petL* | 1185 | 896 | 906 | 0.068 | 5.385164 | 0.011118 |
| *trnS–ycf3* | 966 | 858 | 916 | 0.052 | 4.795831 | 0.010089 |
| *trnT–psbD* | 1322 | 876 | 891 | 0.06 | 5.385164 | 0.008906 |
| *atpI–atpH* | 1166 | 1005 | 1017 | 0.071 | 5.385164 | 0.021976 |
| *rps12–rpl20* | 828 | 786 | 795 | 0.043 | 4.582575 | 0.011161 |
| *petD intron* | 803 | 733 | 738 | 0.034 | 4.582575 | 0.008686 |
| *trnA intron* | 884 | 884 | 884 | 0.025 | 4.123105 | 0.014897 |
| *trnG–trnS* | 852 | 690 | 737 | 0.083 | 5.567764 | 0.024811 |
| *clpP intron 2* | 663 | 627 | 648 | 0.073 | 6.082762 | 0.017736 |
| *rps16 intron* | 863 | 849 | 863 | 0.057 | 6.708203 | 0.012684 |
| *petB intron* | 734 | 709 | 719 | 0.057 | 6.557438 | 0.012649 |
| *ycf3 intron 1* | 734 | 716 | 722 | 0.032 | 5.916079 | 0.010386 |
| *trnC–petN* | 874 | 810 | 829 | 0.069 | 9.219544 | 0.008691 |
| *rpoC1 intron* | 877 | 779 | 807 | 0.059 | 8.831760 | 0.012004 |
| *trnC–rpoB* | 1210 | 1130 | 1141 | 0.048 | 8.774964 | 0.018412 |
| *rbcL–atpB* | 1262 | 773 | 1131 | 0.045 | 9.219544 | 0.012154 |
| *trnI intron* | 1023 | 1005 | 1019 | 0.032 | 7.745966 | 0.020568 |
| *atpF intron* | 724 | 691 | 698 | 0.033 | 8.062257 | 0.010298 |
| *ycf3 intron 2* | 769 | 720 | 732 | 0.048 | 9.273618 | 0.014167 |
| *trnK–matK* | 711 | 695 | 708 | 0.052 | 9.433981 | 0.013678 |
| *ndhJ–trnF* | 744 | 589 | 664 | 0.19 | 8.774964 | 0.118809 |
| *trnR–trnN* | 633 | 632 | 633 | 0.071 | 3.162277 | 0.163359 |
| *ndhB intron* | 679 | 669 | 679 | 0.025 | 13.34166 | 0.022537 |
| *rps12 intron* | 536 | 536 | 536 | 0.007 | 13.26649 | 0.020085 |
